# Association between soft drink, fruit juice consumption and obesity in Eastern Europe: cross‐sectional and longitudinal analysis of the HAPIEE study

**DOI:** 10.1111/jhn.12696

**Published:** 2019-09-01

**Authors:** A. Garduño‐Alanís, S. Malyutina, A. Pajak, U. Stepaniak, R. Kubinova, D. Denisova, H. Pikhart, A. Peasey, M. Bobak, D. Stefler

**Affiliations:** ^1^ Department of Epidemiology and Public Health University College London London UK; ^2^ Research Department Universidad de la Salud del Estado de México Toluca México; ^3^ Research Institute of Internal and Preventive Medicine – Branch of the Institute of Cytology and Genetics Siberian Branch of Russian Academy of Sciences Novosibirsk Russia; ^4^ Novosibirsk State Medical University Novosibirsk Russia; ^5^ Department of Epidemiology and Population Studies Jagiellonian University Collegium Medicum Krakow Poland; ^6^ National Institute of Public Health Prague Czech Republic

**Keywords:** body mass index, Eastern Europe, fruit juice, soft drinks

## Abstract

**Background:**

Fruit juice and soft drink consumption have been shown to be related to obesity. However, this relationship has not been explored in Eastern Europe. The present study aimed to assess the cross‐sectional and longitudinal relationships between fruit juice, soft drink consumption and body mass index (BMI) in Eastern European cohorts.

**Methods:**

Data from the Health, Alcohol and Psychosocial factors in Eastern Europe population‐based prospective cohort study, based in Russia, Poland and the Czech Republic, were used. Intakes of sugar‐sweetened beverage (SSB), artificially‐sweetened beverage (ASB) and fruit juice were estimated from a food frequency questionnaire. Participant BMI values were assessed at baseline (*n* = 26 634) and after a 3‐year follow‐up (data available only for Russia, *n* = 5205).

**Results:**

Soft drink consumption was generally low, particularly in Russia. Compared to never drinkers of SSB, participants who drank SSB every day had a significantly higher BMI in the Czech [β‐coefficient = 0.28; 95% confidence interval (CI) = 0.02–0.54], Russian (β‐coefficient = 1.38; 95% CI = 0.62–2.15) and Polish (β‐coefficient = 0.83; 95% CI = 0.29–1.37) cohorts. Occasional or daily ASB consumption was also positively associated with BMI in all three cohorts. Results for daily fruit juice intake were inconsistent, with a positive association amongst Russians (β‐coefficient = 0.75; 95% CI = 0.28–1.21) but a negative trend in the Czech Republic (β‐coefficient = −0.42; 95% CI = −0.86 to 0.02). Russians participants who drank SSB or ASB had an increased BMI after follow‐up.

**Conclusions:**

Our findings support previous studies suggesting that soft drink consumption (including SSBs and ASBs) is positively related to BMI, whereas our results for fruit juice were less consistent. Policies regarding these beverages should be considered in Eastern Europe to lower the risk of obesity.

## Introduction

Cardiovascular disease (CVD) mortality and morbidity rates in Eastern European countries (EECs) are considerably higher than in the West 1. This health gap emerged in the 1970s, became more pronounced after the political reconstruction in the early 1990s and, despite some reduction in recent years, it still exists today 2.

It is likely that unhealthy diet has contributed to the high CVD rates in EECs 2, 3, 4. For example, the subsidy of foods with a high saturated fat content could have contributed to the excessive consumption of such products before the 1990s 3. More recent data from EEC show that intakes of saturated fats, sugar and meat products are still too high, whereas the consumption of fruits and vegetables is lower than World Health Organization (WHO) recommendations 5, 6. Other diet‐related risk factors, such as obesity, could have also contributed to the health gap between Eastern and Western Europe. Obesity rates have almost tripled over the last 30 years globally 7, 8, and similar trends can be observed in EEC. 9, 10 Projections suggest a considerable further increase in the prevalence of obesity in EECs by 2050 11.

The consumption of soft drinks, including sugar‐sweetened beverages (SSBs) and artificial‐sweetened beverages (ASB), has increased substantially during the past decades in most parts of the world 12. Although Eastern Europeans are considered to be low consumers in this aspect 13, increasing trends can be observed here too, particularly after the political reconstruction in 1990 12. Because most previous studies show that regular soft drink consumption is related to a higher body mass index (BMI), these products may be partly responsible for the global obesity epidemic 14, 15, 16, 17. However, the available evidence is not entirely consistent 18, 19. A similar debate has emerged regarding fruit juice consumption 20, 21. Although moderate fruit juice intake may provide nutritional benefits and does not appear to have a negative impact on body weight measures, 20 some studies have shown that their regular intake was positively associated with long‐term weight gain 21.

Consumption patterns of soft drinks and fruit juices have not been explored in Eastern European adults, and their association with obesity within this region has not been assessed. Using data from the Health, Alcohol and Psychosocial Factors in Eastern Europe (HAPIEE) prospective cohort study, we examined the cross‐sectional relationship between fruit juice/soft drink consumption and obesity in Russian, Czech and Polish cohorts, and we explored whether these drinks affect BMI change over time in the Russian cohort where follow‐up data were available.

## Materials and methods

### Study sample

The HAPIEE study is a multicentre prospective cohort study with participants recruited in Russia, Poland and Czech Republic 22. The cohorts in each country consisted of random samples of men and women aged 45–69 years at baseline, who were selected from population registers in Novosibirsk (Russia), Krakow (Poland) and six towns in the Czech Republic, stratified by gender and 5‐year age groups. The overall response rate was 59% 22.

From 28 945 participants at baseline, those who had missing data on the exposure (*n* = 718), outcome (*n* = 46) and covariates (*n* = 1283) were excluded from the sample. Individuals with extreme values for weight (more than 200 kg), height (more than 205 cm) and energy intake (more than 5000 kcal day^−1^ or less than 500 kcal day^−1^) were also excluded (*n* = 264). After these exclusions, the analytical sample for the cross‐sectional assessment consisted of 26 634 individuals. In addition to the cross‐sectional analysis with baseline measurements, BMI change over time was assessed in the Russian cohort. From the 6182 individuals who participated in the second wave of the study in this country, data on height and weight measurements were available for 5205 people.

### Data collection

Baseline survey (wave 1) was conducted between 2002 and 2005. In Russia, questionnaires and examinations were carried out in a clinic. In Poland and the Czech Republic, questionnaires were completed at home and examinations were carried out in a clinic. The structured questionnaires covered health, lifestyle, food frequency, socioeconomic circumstances, psychosocial factors and psychosocial environment at work. The examination included anthropometric, physical, cognitive and blood evaluations. The cohorts were re‐interviewed in 2006–2008 (wave 2) 22, although height and weight were measured only in Russian participants; therefore, longitudinal evaluations analyses could not be performed in the Polish and Czech cohorts.

Participants were asked ‘how often, on average in the last 3 months they consumed specific foods and drinks’, details of this dietary data collection procedures with food frequency questionnaires (FFQ) have been described earlier 23. The FFQ item that asked the participants about the intake of non‐alcoholic carbonated (fizzy) drinks, such as coke, fizzy orange or lemonade, was used to estimate SSB consumption, whereas the item on low‐calorie (diet) carbonated drinks was used for ASB. Fruit juice intake was also assessed with one FFQ item which asked about the intake of fruit juices, such as apple drinks. For all fruit juice and soft drinks, one drink was equivalent to 200 mL. For the current analysis, all participants were classified into three categories of their SSB, ASB or fruit juice consumption: never drinkers, occasional drinkers (less than one drink per day) and daily drinkers (one or more drinks per day). Three categories were specified to assess the gradient in the potential effect and not just the difference between drinkers and nondrinkers.

Measured and self‐reported height and weight were used to calculate the BMI. Height was measured using a mechanical stadiometer and weight was assessed with an electronic scale (both measurements were obtained without shoes and outer clothes) 23. At baseline, 3085 (10.7%) participants had missing data on either measured weight or measured height; to avoid losing so many subjects, missing data of measured weight and height were replaced by self‐reported weight and height, respectively. This replacement was based on a high correlation between the measured and self‐reported data in participants with both indicators available (*r* = 0.97, *r* = 0.95). For further confirmation, we also ran the analysis on participants with measured BMI values, and the results obtained were similar to our main findings. BMI was calculated dividing body weight by the square of body height (kg m^–2^). In line with the WHO categorisation of BMI for adult population, obesity was defined as BMI ≥30 kg m^–2^.

In the second wave of data collection in the Russian cohort, measured height and weight was used to calculate BMI. The change in BMI was obtained by subtracting BMI at baseline from BMI in wave 2.

### Ethical approval

All participants provided informed consent prior to their inclusion in the study. Study protocols were approved by ethical committees at University College London, and all participating centres in Poland, Russia and the Czech Republic and have therefore been performed in accordance with the ethical standards laid down in the 1964 Declaration of Helsinki and its later amendments.

### Statistical analysis

The cross‐sectional associations between the exposure (fruit juice, SSB and ASB consumption) and outcome (BMI) variables were assessed with multivariable‐adjusted linear and logistic regression models. In the linear regression, BMI was used as continuous variable, whereas, in the logistic regression models, BMI was dichotomised in two categories: obese (BMI ≥30 kg m^–2^) and non‐obese (BMI <30 kg m^–2^). All associations were assessed in three models. In model 1, these were adjusted for age and sex. In model 2, they were further adjusted for socio‐demographic variables, such as education and marital status. Finally, in model 3, lifestyle factors that can act as potential confounders, including smoking, alcohol consumption, physical activity, energy intake and fruit and vegetable consumption, as well as previously diagnosed chronic diseases, such as diabetes, CVD or cancer, were also included.

Because we found statistically significant heterogeneity in country cohort‐specific associations of SSB, ASB and fruit juice consumption with BMI (*P *< 0.001), all results are presented separately in the Czech, Polish and Russian samples, and pooled results are not shown. No other covariates emerged as significant effect modifiers across the three cohorts.

In the longitudinal analysis, associations between the exposure (fruit juice, SSB and ASB) and changes in BMI were examined using multivariable logistic regression models. For this analysis, an increase in BMI of more than 1 kg m^–2^ between wave 1 and 2 was used as the main outcome variable. As a result of the low number of daily soft drink consumers and the consequent impact on statistical power, in this part of the analysis, we compared only two categories of participants: drinkers and nondrinkers.

All tests were performed with stata, version 15 (StataCorp, College Station, TX, USA). *P* < 0.05 was considered statistically significant.

## Results

Descriptive characteristics of the sample are shown in Table 1. The mean BMI in the analytical sample was 28 kg m^–2^ or higher in all three cohorts. With the exception of SSB intake among Czech participants, soft drink consumption was generally low, particularly in Russia, where the prevalence of daily SSB and ASB consumption was <2% and 1%, respectively. Fruit juice consumption was also found to be relatively low, with less than 10% of the sample reported to drink it every day in Russia and the Czech Republic.

**Table 1:** Descriptive characteristics of the study sample by country (*n* = 26 634)

Variable	Country			
	Czech (*n* = 7741)	Russia (*n* = 9218)	Poland (*n* = 9675)	Total (*n* = 26 634)
BMI (kg m^–2^), mean (SD)	28.1 (4.5)	28.5 (5.5)	28.0 (4.6)	28.2 (5)
Fruit juice consumption, *n* (%)				
Never drink	3503 (45.2)	3672 (39.8)	2477 (25.6)	9652 (36.2)
<1 per day	3796 (49.0)	4992 (54.2)	5456 (56.4)	14 244 (53.5)
≥1 per day	442 (5.7)	554 (6.0)	1742 (18.0)	2738 (10.3)
Soft drinks				
SSB consumption, *n* (%)				
Never drink	3621 (46.8)	7142 (77.5)	7935 (82.0)	18 698 (70.2)
<1 per day	2386 (30.8)	1903 (20.6)	1468 (15.2)	5757 (21.6)
≥1 per day	1734 (22.4)	173 (1.9)	272 (2.8)	2179 (8.2)
ASB consumption, *n* (%)				
Never drink	5697 (73.6)	8961 (97.2)	8156 (84.3)	22 814 (85.7)
<1 per day	1480 (19.1)	205 (2.2)	908 (9.4)	2593 (9.7)
≥1 per day	564 (7.3)	52 (0.6)	611 (6.3)	1227 (4.6)
Age group (years), *n* (%)				
<55	2913 (37.6)	3341 (36.2)	3793 (39.2)	10 047 (37.7)
55–65	3212 (41.5)	3724 (40.4)	3950 (40.8)	10 886 (40.9)
>65	1616 (20.9)	2153 (23.4)	1932 (20.0)	57.01 (21.4)
Sex, *n* (%)				
Men	3642 (47.0)	4164 (45.2)	4703 (48.6)	12 509 (47.0)
Woman	4099 (53.0)	5054 (54.8)	4972 (51.4)	14 125 (53.0)
Education, *n* (%)				
Primary or less	900 (11.6)	967 (10.5)	1124 (11.6)	2991 (11.2)
Vocational	2802 (36.2)	2445 (26.5)	2034 (21.0)	7281 (27.3)
Secondary	2912 (37.6)	3146 (34.1)	3748 (38.7)	9806 (36.8)
University degree	1127 (14.6)	2660 (28.9)	2769 (28.6)	6556 (24.6)
Marital status, *n* (%)				
Living with a partner	5903 (76.3)	6649 (72.1)	7384 (76.3)	19 936 (74.8)
Living without a partner	1838 (23.7)	2569 (27.9)	2291 (23.7)	6698 (25.2)
Smoking, *n* (%)				
Never smoker	3370 (43.5)	5377 (58.3)	3832 (39.6)	12 579 (47.2)
Ex‐smoker	2315 (30.0)	1255 (13.6)	2733 (28.3)	6303 (23.7)
Regular smoker	2056 (26.6)	2586 (28.1)	3110 (32.1)	7752 (29.1)
Alcohol consumption (g day^−1^), *n* (%)				
0	921 (11.9)	1472 (16.0)	3289 (34.0)	5682 (21.3)
>0–20	5494 (71.0)	6630 (72.0)	5797 (60.0)	17 921 (67.3)
>20	1326 (17.1)	1116 (12.1)	589 (6.1)	3031 (11.4)
Physical activity (MET‐h day^−1^), *n* (%)				
<5	2711 (35.0)	2638 (28.6)	2861 (29.6)	8210 (30.8)
5–15	3809 (49.2)	5245 (56.9)	5083 (52.5)	14 137 (53.1)
>15	1221 (15.8)	1335 (14.5)	1731 (17.9)	4287 (16.1)
Energy (kcal day^−1^), *n* (%)				
<2000	4320 (55.8)	2419 (26.2)	4351 (45.0)	11 090 (41.7)
2000–2500	1884 (24.3)	2487 (27.0)	2857 (29.5)	7228 (27.1)
>2500	1537 (19.9)	4312 (46.8)	2467 (25.5)	8316 (31.2)
Fruits and vegetables consumption (g day^−1^), *n* (%)				
<300	1825 (23.6)	3531 (38.3)	2500 (25.8)	7856 (29.5)
300–600	3064 (39.6)	4154 (45.1)	4485 (46.4)	11 703 (44.0)
>600	2852 (36.8)	1533 (16.6)	2690 (27.8)	7075 (26.6)
CVD or cancer in medical history, *n* (%)				
No	6664 (86.1)	7808 (84.7)	8196 (84.7)	22 668 (85.1)
Yes	1077 (13.9)	1410 (15.3)	1479 (15.3)	3966 (14.9)
Diabetes in medical history, *n* (%)				
No	6863 (88.7)	8728 (95.7)	8571 (88.6)	24 162 (90.7)
Yes	878 (11.3)	490 (5.3)	1104 (11.4)	2472 (9.3)

ASB, artificially sweetened beverages; BMI, body mass index; CVD, cardiovascular disease; MET, metabolic equivalents; SSB, sugar‐sweetened beverages.

In the bivariate analysis (Table 2), almost all of the covariates were associated with fruit juice, SSB and ASB consumption in the pooled sample. Daily fruit juice consumption was found to be considerably more common in females, participants with higher education and abstainers from alcohol, as well as among those who reported higher physical activity. By contrast, regular SSB intake was more common in males, individuals with lower education and regular alcohol drinkers, as well as in those who reported less exercise. Both SSB and ASB were more common in younger compared to older participants, as well as among regular smokers and among those who eat high amounts of fruits and vegetables. These results were largely similar if the associations were examined separately in the three countries (data not shown).

**Table 2:** Bivariate analysis of main exposure and covariates

	Total *n* = 26 634 (%)	Fruit juice consumption			*P* value† (*P* for trend‡)	SSB consumption			*P* value† (*P* for trend‡)	ASB consumption			*P* value† (*P* for trend‡)
		Never drink, *n* = 9652 (%)	<1 per day, *n* = 14 244 (%)	≥1 per day, *n* = 2738 (%)		Never drink, *n* = 18698 (%)	<1 per day, *n* = 5757 (%)	≥1 per day, *n* = 2179 (%)		Never drink, *n* = 228 14 (%)	<1 per day, *n* = 2593 (%)	≥1 per day, *n* = 1227 (%)	
**Covariates**													
Socio‐demographics													
Age (years)*													
<55	10 047 (100)	27.4	59.8	12.8	<0.001 (<0.001)	63.2	26.9	9.9	<0.001 (<0.001)	83.3	11.7	5.0	<0.001 (0.004)
55–65	10 886 (100)	38.2	52.0	9.8		72.5	19.9	7.6		86.3	9.1	4.6	
>65	5701 (100)	48.1	45.1	6.8		78.1	15.6	6.3		88.5	7.5	4.0	
Sex													
Men	12 509 (100)	38.0	53.4	8.6	<0.001	63.9	26.2	9.9	<0.001	84.2	11.0	4.7	<0.001
Women	14 125 (100)	34.7	53.6	11.7		75.7	17.6	6.7		87.0	8.6	4.5	
Education*													
Primary or less	2991 (100)	51.7	42.2	6.1	<0.001 (<0.001)	70.9	19.9	9.2	<0.001 (<0.001)	85.0	10.0	5.0	<0.001 (<0.001)
Vocational	7281 (100)	40.4	51.5	8.1		65.0	22.5	12.5		83.4	11.2	5.4	
Secondary	9806 (100)	35.3	54.7	10.0		70.2	22.7	7.1		85.9	9.5	4.6	
University degree	6556 (100)	26.0	59.1	14.9		75.6	19.9	4.5		88.1	8.3	3.6	
Marital status													
Living with a partner	19 936 (100)	35.0	54.5	10.5	<0.001	68.7	22.7	8.7	<0.001	84.9	10.3	4.8	<0.001
Living alone	6698 (100)	39.9	50.5	9.6		74.8	18.5	6.7		88.0	8.2	3.8	
Lifestyle behaviours/medical history													
Smoking													
Never smoker	12 579 (100)	34.9	55.3	9.8	<0.001	73.3	20.2	6.5	<0.001	87.7	8.5	3.8	<0.001
Ex‐smoker	6303 (100)	37.5	51.8	10.7		69.5	21.2	9.3		82.7	11.4	5.9	
Regular smoker	7752 (100)	37.4	51.8	10.8		65.7	24.2	10.1		84.8	10.3	4.9	
Alcohol consumption (g day^−1^)*													
0	5682 (100)	39.7	48.7	11.6	<0.001 (<0.001)	81.2	13.9	4.9	<0.001 (<0.001)	86.5	7.7	5.8	<0.001 (<0.001)
>0–20	17 921 (100)	34.2	55.5	10.3		69.0	22.6	8.4		85.7	10.0	4.3	
>20	3031 (100)	42.0	50.5	7.5		56.7	30.5	12.8		84.1	11.6	4.3	
Physical activity (MET‐h day^−1^)*													
<5	8210 (100)	38.9	51.2	9.9	<0.001 (<0.01)	67.5	22.4	10.1	<0.001 (<0.01)	85.6	10.2	4.2	<0.001 (<0.001)
5–15	14 137 (100)	34.5	55.4	10.1		70.6	21.9	7.5		85.9	9.7	4.4	
>15	4287 (100)	36.9	51.5	11.6		74.2	19.2	6.6		84.9	8.8	6.3	
Energy (kcal day^−1^)*													
<2000	11 090 (100)	43.3	50.3	6.5	<0.001 (<0.001)	74.0	18.9	7.1	<0.001 (<0.001)	85.1	10.1	4.8	0.222 (0.530)
2000–2500	7228 (100)	34.6	54.9	11.0		70.6	21.5	7.9		85.2	9.9	4.4	
>2500	8316 (100)	28.3	56.9	14.8		64.8	25.3	9.9		86.2	9.2	4.6	
Fruits and vegetables consumption (g day^−1^)*													
<300	7856 (100)	47.3	47.7	5.0	<0.001 (<0.001)	69.4	23.2	7.4	<0.001 (<0.001)	89.5	7.6	2.9	<0.001 (<0.001)
300–600	11 703 (100)	33.0	57.2	9.8		71.4	21.0	7.6		85.9	9.5	4.6	
>600	7075 (100)	29.3	53.7	17.0		69.1	20.8	10.1		81.1	12.5	6.4	
CVD or cancer in medical history													
No	22 668 (100)	35.3	54.4	10.4	<0.001	69.2	22.4	8.4	<0.001	85.7	9.8	4.5	<0.001
Yes	3966 (100)	41.9	48.4	9.7		75.7	17.4	6.9		85.7	9.2	5.1	
Diabetes in medical history													
No	24 162 (100)	34.8	54.7	10.5	<0.001	68.9	22.5	8.6	<0.001	86.5	9.4	4.1	<0.001
Yes	2472 (100)	50.7	41.3	8.0		83.0	13.1	3.9		78.0	12.8	9.2	

ASB, artificially sweetened beverages; CVD cardiovascular disease; MET, metabolic equivalents; SSB, sugar‐sweetened beverages.

* To test χ^2^ for trend, the main exposure variables were re‐categorised in a binary outcome: (never drink + <1 per day) versus (≥1 per day).

† χ^2^.

‡ χ^2^ for trend. *P* < 0.05 was considered statistically significant.

Table 3 shows the results of the multivariable‐adjusted linear regression analysis for the association of fruit juice, SSB and ASB consumption with BMI, separately by country‐cohorts.

**Table 3:** Multivariable linear regression for body mass index and fruit juice/soft drink consumption, by country

Country	Exposure	Intake level	*n*	Model 1			Model 2			Model 3		
				β coeff.	95% CI	*P* value	β coeff.	95% CI	*P* value	β coeff.	95% CI	*P* value
Czech	Fruit juice	Never	3505	ref.			ref.			ref.		
		<1 day^−1^	3796	−0.68	−0.89, −0.47	<0.001	−0.49	−0.70, −0.28	<0.001	−0.30	−0.50, −0.09	0.005
		≥1 day^−1^	442	−0.83	−1.28, −0.39	<0.001	−0.69	−1.17, −0.24	0.002	−0.42	−0.86, 0.02	0.060
	SSB	Never	3621	ref.			ref.			ref.		
		<1 day^−1^	2386	−0.16	−0.39, 0.08	0.195	−0.14	−0.37, 0.09	0.233	0.20	−0.02, 0.44	0.081
		≥1 day^−1^	1734	−0.02	−0.28, 0.24	0.903	−0.21	−0.47, 0.04	0.103	0.28	0.02, 0.54	0.037
	ASB	Never	5697	ref.			ref.			ref.		
		<1 day^−1^	1480	0.57	0.31, 0.82	<0.001	0.57	0.32, 0.82	<0.001	0.48	0.23, 0.73	<0.001
		≥1 per day	564	1.82	1.43, 2.20	<0.001	1.70	1.32, 2.08	<0.001	1.47	1.09, 1.84	<0.001
Russia	Fruit juice	Never	3672	ref.			ref.			ref.		
		<1 per day	4992	0.14	−0.09, 0.36	0.236	0.18	−0.05, 0.40	0.127	0.13	−0.10, 0.35	0.267
		≥1 per day	554	0.65	0.18, 1.11	0.007	0.77	0.30, 1.25	0.001	0.75	0.28, 1.21	0.002
	SSB	Never	7142	ref.			ref.			ref.		
		<1 per day	1903	0.48	0.15, 0.68	0.002	0.33	0.06, 0.59	0.015	0.49	0.23, 0.75	<0.001
		≥1 per day	173	1.39	0.61, 2.17	<0.001	1.31	0.53, 2.09	0.001	1.38	0.62, 2.15	<0.001
	ASB	Never	8961	ref.			ref.			ref.		
		<1 per day	205	0.99	0.28, 1.71	0.007	0.98	0.27, 1.70	0.007	0.74	0.04, 1.44	0.039
		≥1 per day	52	−0.01	−1.42, 1.40	0.985	−0.02	−1.42, 1.38	0.978	−0.27	−1.64, 1.11	0.704
Poland	Fruit juice	Never	2477	ref.			ref.			ref.		
		<1 per day	5456	−0.05	−0.27, 0.17	0.629	0.12	−0.10, 0.34	0.299	0.20	−0.02, 0.41	0.074
		≥1 per day	1742	−0.06	−0.34, 0.23	0.685	0.21	−0.08, 0.50	0.153	0.25	−0.03, 0.54	0.082
	SSB	Never	7935	ref.	ref.	ref.	ref.	ref.	ref.	ref.	ref.	ref.
		<1 per day	1468	0.21	−0.05, 0.48	0.114	0.21	−0.05, 0.48	0.108	0.41	0.16, 0.67	0.001
		≥1 per day	272	0.58	0.02, 1.14	0.042	0.53	−0.03, 1.08	0.062	0.83	0.29, 1.37	0.003
	ASB	Never	8156	ref.	ref.	ref.	ref.	ref.	ref.	ref.	ref.	ref.
		<1 per day	908	0.26	−0.06, 0.58	0.109	0.21	−0.10, 0.53	0.19	0.30	0.00, 0.61	0.051
		≥1 per day	611	0.80	0.42, 1.18	<0.001	0.80	0.38, 1.14	<0.001	0.62	0.26, 0.99	0.001

ASB, artificially sweetened beverages; CI, confidence interval; coeff., coefficient; ref., reference; SSB, sugar‐sweetened beverages.

Model 1: Adjusted for: age + sex. Model 2: model 1 + education + marital status. Model 3: Model 2 + smoking + alcohol consumption + physical activity + energy consumption + fruits and vegetables consumption + cardiovascular disease or cancer in medical history + diabetes in medical history. *P* < 0.05 was considered statistically significant.

Participants who drank fruit juice every day had significantly higher BMI compared to never drinkers in the Russian sample, and a similar positive trend, although statistically not significant, was found in Poland. However, the direction of the association was the opposite in the Czech cohort, indicating lower BMI among daily fruit juice drinkers with borderline statistical significance after multivariable adjustment.

Regarding SSB, we found a positive association with a clear dose–response gradient across occasional and daily drinkers in all three cohorts. Compared to never drinkers, individuals with occasional or regular ASB intake had a significantly higher BMI in the Czech and Polish cohorts, whereas this positive association was statistically significant among occasional drinkers in Russia.

Results were similar when the associations were examined with logistic regression models using obesity (BMI > 30 kg m^–2^), as the main outcome variable (*n* = 8358) (see Supporting information, Table S1).

Table 4 shows the results of the multivariable logistic regression models for the association between fruit juice/soft drink consumption and an increase in BMI of more than 1 kg m^–2^ over an average follow‐up of 3 years among Russian participants. BMI increased by more than 1 kg m^–2^ in 1789 participants (34.4% of the sample), whereas it decreased or increased <1 kg m^–2^ in 3416 people (65.6%). The mean (SD) change of BMI in these groups was 2.4 (2.6) kg m^–2^ and −0.56 (1.3) kg m^–2^, respectively. We found that SSB and ASB intake was significantly related to BMI increase. On the other hand, fruit juice consumption was associated with lower risk of BMI increase but this association was not statistically significant.

**Table 4:** Multivariable logistic regression for a unit change in body mass index and fruit juice/soft drink consumption, at second wave (2006–2008) in the cohort in Russia (*n* = 5205)

Exposure	Intake level	*n*	Model 1			Model 2			Model 3		
			OR	95% CI	*P* value	OR	95% CI	*P* value	OR	95% CI	*P* value
Fruit juice	No Drinkers	1963	ref.			ref.			ref.		
	Drinkers	3242	0.90	0.80, 1.02	0.106	0.94	0.83, 1.07	0.368	0.92	0.81, 1.05	0.203
SSB	No Drinkers	4048	ref.			ref.			ref.		
	Drinkers	1157	1.28	1.11, 1.47	<0.001	1.25	1.09, 1.44	0.001	1.26	1.09, 1.45	0.001
ASB	No Drinkers	5091	ref.			ref.			ref.		
	Drinkers	114	1.75	1.21, 2.55	0.003	1.75	1.20, 2.54	0.004	1.63	1.12, 2.39	0.012

1

Body mass index (BMI) change ≥ 1 units (*n* = 1789; 34.4%) vs. BMI change < 1 units (*n* = 3416; 65.6%). Model 1: Adjusted for: age + sex. Model 2: model 1 + education + marital status. Model 3: Model 2 + smoking + alcohol consumption + physical activity + energy consumption + fruits and vegetables consumption + BMI at baseline + cardiovascular disease or cancer in medical history + diabetes in medical history. *P* < 0.05 was considered statistically significant. 1 unit change = 1 kg m^–2^.

## Discussion

### Main findings

In the present study investigating soft drink and fruit juice consumption in three Eastern European cohorts, we found a relatively low prevalence of daily consumption of both, particularly in Russia. Despite some inconsistencies across cohorts, the cross‐sectional analyses indicated that occasional or daily SSB and ASB consumptions were related to higher BMI. The prospective analysis of the Russian cohort also suggested that individuals who occasionally drank these food products had a higher risk of increased BMI at follow‐up. The results on fruit juice consumption were inconsistent because the BMI of regular drinkers, compared to never drinkers, appeared to be higher in the Russia and Poland but lower in the Czech Republic.

### Interpretation of the results

Overall, 10.3% of the participants reported that they drank fruit juice every day, which is lower than the intakes reported for Western European countries. 16 The observed rates of daily soft drink consumption of 8.2% and 4.6% for SSB and ASB, respectively, are low compared to global reports from 2016 13, 16 . However, the mean age of our respondents was 58 years, and drinking of SSB is much more common at a younger age. For example, everyday consumption of fruit juice was observed in 28% and SBB in 17% of young people in Poland. 24 Furthermore, because the data for the present study were collected in 2002–2004, consumption habits may have subsequently changed. Nevertheless, more recent surveys also suggest a relatively low intake of sugary drinks in Russia 25.

Our results for the association between soft drinks, fruit juice consumption and BMI are generally consistent with existing literature. Previously published studies that examined the link between fruit juice intake and obesity often produced conflicting results 26, 27. Although fruit juice consumption has been associated with a small amount of long‐term weight gain 28, a moderate amount of fruit juice could be recommended to different populations without detrimental effects on weight 20. Because the sugar content of fruit juice is similar or higher than those of whole fruits, whereas it contains much less fibres, its beneficial effect on health is probably weaker compared with fruits 29. In the present study, the conflicting results between the Czech and Russian cohorts may be also explained by residual confounding, such as a stronger link between fruit juice intake and a health‐conscious lifestyle in the Czech Republic.

Regarding SSB, we found that occasional or everyday consumption was associated with higher BMI in all three cohorts. This result is consistent with previous evidence obtained from other populations, as supported by both observational studies and randomised controlled trials 14, 30, 31. For example, in a recent systematic review, among 26 observational studies, only one reported no association between SSB intake and weight gain 30.

In terms of possible mechanism, SSB consumption can lead to weight gain either directly, through higher energy intake from the drinks themselves, or indirectly through energy intake from other food products, because calorie intake from liquid carbohydrates could result in less satiety 32.

Despite the relatively consistent literature, the strength of the relationship between SSB consumption and obesity, as well as the independence of this association from potential confounding factors, is difficult to establish 33. To overcome the methodological limitations inherent in observational studies and strengthen the evidence further, high‐quality randomised controlled trials with adequate design and sample size are clearly warranted 33.

In our analysis, the most consistent positive relationship with obesity was found for ASB intake, also known as diet sodas. As opposed to SSBs, which contain added caloric sweeteners, such as sucrose, high‐fructose corn syrup or fruit‐juice concentrates, ASBs contain nonsugar sweeteners 34. Several previous studies on ASB consumption are in accordance with our findings. For example, a higher intake of ASB was found to be related to increased body fat in UK children 35. Among adults, previous studies found positive relationships of ASB consumption with BMI, abdominal obesity and metabolic syndrome 36, 37, 38. In addition, there is some evidence that increased BMI may play a role in the link between ASB intake and the risk of diabetes 36. However, the available evidence is inconsistent, and there are several potential explanations for the observed positive associations between ASB intake and obesity. 33 These may include (i) reverse causation, meaning that people tend to drink ASB instead of SSB when they have obesity 38; (ii) an increase in sweet preference and appetite associated with ASB consumption 39; and (iii) common artificial sweeteners used in ASB, which could generate a similar body response in terms of satiety compared to SSB 40. Therefore, future studies that examine the metabolic effects of ASB are still needed.

The results of our longitudinal analysis in Russia are similar to what has been observed in other prospective studies. For example, an increase in body weight of 4–5 kg was found in women whose SSB consumption changed from occasional to everyday over 4 years of follow‐up 41. Another US‐based cohort study indicated significant increase in the risk of obesity and overweight over time among those who consumed ASB every day 17, 39.

### Limitations and strengths

The present study has several limitations that need to be taken into account when interpreting the results.

First, the cross‐sectional design of our study does not allow a clear interpretation of temporality, and reverse causation may play a role in some of the observed relationships. For example, people with obesity might reduce their soft drink consumption leading to potential misinterpretation of the main association. Reverse causation might be also the plausible explanation for the observed positive association between ASB intake and obesity. However, the fact that the longitudinal assessment of BMI change over time provided similar results may serve as internal validation and makes this possibility less likely.

Second, the measurement of fruit juice and soft drink intake by FFQ is likely to be imprecise, and this may lead to misclassification of these exposures and inaccurate estimates of the associations. The FFQ is a common method for assessing dietary patterns in epidemiology, although it has been criticised as being imprecise and affected by information bias 42. Self‐reported measures of fruit juice are prone to under‐ 36 and over‐reporting 43, and soft drink consumption is prone to under‐reporting 30. The validity of the dietary data in HAPIEE study was tested using biomarkers regarding fruit and vegetable consumption. However, no such assessment was possible for fruit juice and soft drinks 44. Similarly, self‐reported weight and height were also prone to misclassification of BMI. However, the high completeness of objective measurement and the high correlation between self‐reported and measured weight and height make this bias less likely.

Third, the moderate response rates and urban character of the HAPIEE cohorts make is impossible to generalise the findings to the whole population. It is also likely that responders were healthier compared to the general population. However, these issues should not affect the internal validity of our results.

Fourth, although the large sample size is an important strength of the present study, the fact that only a small proportion of participants consumed fruit juice and soft drinks on a regular basis reduces the statistical power of the analysis. This can lead to wide confidence intervals and may be the reason for some of the observed nonsignificant associations.

Finally, the residual confounding is inherent to the observational study design. Nevertheless, the multiple adjustment for several socio‐economic and lifestyle variables reduce this possibility.

The main strengths of the present study are that this is one of the largest cohort studies to investigate the relationship between fruit juice/soft drink consumption and BMI in Eastern Europe. It is also important that the Russian cohort has a longitudinal element allowing assessment of the role of fruit juice/soft drink consumption in BMI change.

## Conclusions

Our findings support the hypothesis that soft drink consumption, including both SSBs and ASBs, is positively related to BMI and may lead to obesity. However, the findings regarding the role of fruit juice were inconsistent. Policies regarding soft drink beverages may need to be considered in Eastern Europe to reduce the rates of obesity in the region.

**Conflict of interests, source of funding and authorship**

The authors declare that they have no conflicts of interest.

The HAPIEE study was funded by the Wellcome Trust (grant numbers WT064947, WT081081), the US National Institute of Aging (grant number 1RO1AG23522) and the MacArthur Foundation Initiative on Social Upheaval and Health.

AG‐A took part in the concept and design of the analysis, carried out the statistical analysis, interpreted the results and drafted the manuscript. SM, AP and RK contributed to the design of the study, led the field work and critically revised the manuscript. US and DD contributed to the data collection and analysis, and critically revised the manuscript. HP, AP and MB contributed to the conception and design of the HAPIEE study, supported the statistical analyses and critically revised the manuscript. DS contributed to the conception and design of the analysis, supported the statistical analysis, helped to interpret the results and critically revised subsequent drafts of the manuscript. All authors critically reviewed the manuscript and approved the final version submitted for publication.

## Transparency declaration

The lead author affirms that this manuscript is an honest, accurate and transparent account of the study being reported. The reporting of this work is compliant with STROBE guidelines. The lead author affirms that no important aspects of the study have been omitted and that any discrepancies from the study as planned have been explained.

## Supporting information

**Table S1**. Multivariable logistic regression for body mass index and fruit juice/soft drink consumption by country.

## Footnotes

1 ASB, artificially sweetened beverages; CI, confidence interval; coeff., coefficient; ref., reference; SSB, sugar‐sweetened beverages.
