# Supplementary material for: Association between soft drink, fruit juice consumption and obesity in Eastern Europe: cross‐sectional and longitudinal analysis of the HAPIEE study
Source: J Hum Nutr Diet. 2019 Sep 1;33(1):66–77. doi: 10.1111/jhn.12696 (PMC8425279; doi:10.1111/jhn.12696)
Supplement: Supplementary file 1 — Table S1. Multivariable logistic regression for body mass index and fruit juice/soft drink consumption by country. [file JHN-33-66-s001.docx]

**Table S1. Multivariable logistic regression for BMI and fruit juice/soft drinks consumption by country.**

| **Country** | **Exposure** | **Intake level** | **n** | **Model 1** | | | **Model 2** | | | **Model 3** | | |
| --- | --- | --- | --- | --- | --- | --- | --- | --- | --- | --- | --- | --- |
|  |  |  |  | **β coeff.** | 95% CI | p value | **β coeff.** | 95% CI | p value | **β coeff.** | 95% CI | p value |
| **Czech** | **Fruit juice** | Never | 3505 | **ref.** |  |  | **ref.** |  |  | **ref.** |  |  |
|  |  | <1/day | 3796 | 0.74 | 0.67, 0.82 | <0.001 | 0.80 | 0.72, 0.89 | <0.001 | 0.86 | 0.77, 0.96 | 0.008 |
|  |  | ≥1 per day | 442 | 0.71 | 0.56, 0.87 | 0.003 | 0.75 | 0.59, 0.94 | 0.013 | 0.82 | 0.65, 1.04 | 0.100 |
|  | **SSB** | Never | 3621 | **ref.** |  |  | **ref.** |  |  | **ref.** |  |  |
|  |  | <1/day | 2386 | 0.95 | 0.84, 1.06 | 0.356 | 0.95 | 0.85, 1.07 | 0.413 | 1.10 | 0.97, 1.25 | 0.121 |
|  |  | ≥1 per day | 1734 | 0.92 | 0.81, 1.05 | 0.201 | 0.85 | 0.74, 0.97 | 0.014 | 1.03 | 0.89, 1.18 | 0.689 |
|  | **ASB** | Never | 5697 | **ref.** |  |  | **ref.** |  |  | **ref.** |  |  |
|  |  | <1/day | 1480 | 1.31 | 1.16, 1.49 | <0.001 | 1.32 | 1.16, 1.50 | <0.001 | 1.28 | 1.13, 1.46 | <0.001 |
|  |  | ≥1 per day | 564 | 2.05 | 1.71, 2.45 | <0.001 | 1.98 | 1.65, 2.36 | <0.001 | 1.84 | 1.53, 2.22 | <0.001 |
| **Russia** | **Fruit juice** | Never | 3672 | **ref.** |  |  | **ref.** |  |  | **ref.** |  |  |
|  |  | <1/day | 4992 | 1.06 | 0.97, 1.17 | 0.205 | 1.09 | 0.99, 1.20 | 0.093 | 1.08 | 0.98, 1.19 | 0.141 |
|  |  | ≥1 per day | 554 | 1.13 | 0.93, 1.36 | 0.229 | 1.19 | 0.98, 1.45 | 0.077 | 1.20 | 0.98, 1.47 | 0.073 |
|  | **SSB** | Never | 7142 | **ref.** |  |  | **ref.** |  |  | **ref.** |  |  |
|  |  | <1/day | 1903 | 1.07 | 0.96, 1.20 | 0.215 | 1.04 | 0.93, 1.16 | 0.524 | 1.09 | 0.97, 1.23 | 0.133 |
|  |  | ≥1 per day | 173 | 1.61 | 1.16, 2.23 | 0.004 | 1.56 | 1.13, 2.17 | 0.007 | 1.62 | 1.16, 2.26 | 0.004 |
|  | **ASB** | Never | 8961 | **ref.** |  |  | **ref.** |  |  | **ref.** |  |  |
|  |  | <1/day | 205 | 1.46 | 1.09, 1.97 | 0.012 | 1.46 | 1.08, 1.97 | 0.013 | 1.40 | 1.04, 1.90 | 0.029 |
|  |  | ≥1 per day | 52 | 0.93 | 0.51, 1.70 | 0.805 | 0.92 | 0.50, 1.68 | 0.781 | 0.88 | 0.48, 1.64 | 0.695 |
| **Poland** | **Fruit juice** | Never | 2477 | **ref.** |  |  | **ref.** |  |  | **ref.** |  |  |
|  |  | <1/day | 5456 | 0.92 | 0.83, 1.02 | 0.126 | 0.99 | 0.89, 1.11 | 0.920 | 1.05 | 0.94, 1.17 | 0.387 |
|  |  | ≥1 per day | 1742 | 0.87 | 0.76, 1.00 | 0.044 | 0.98 | 0.85, 1.13 | 0.764 | 1.02 | 0.88, 1.18 | 0.793 |
|  | **SSB** | Never | 7935 | **ref.** |  |  | **ref.** |  |  | **ref.** |  |  |
|  |  | <1/day | 1468 | 1.08 | 0.95, 1.23 | 0.230 | 1.08 | 0.95, 1.23 | 0.223 | 1.18 | 1.04, 1.35 | 0.012 |
|  |  | ≥1 per day | 272 | 1.43 | 1.11, 1.86 | 0.006 | 1.41 | 1.08, 1.83 | 0.010 | 1.60 | 1.22, 2.09 | 0.001 |
|  | **ASB** | Never | 8156 | **ref.** |  |  | **ref.** |  |  | **ref.** |  |  |
|  |  | <1/day | 908 | 1.17 | 1.01, 1.36 | 0.040 | 1.15 | 0.99, 1.34 | 0.073 | 1.20 | 1.03, 1.41 | 0.020 |
|  |  | ≥1 per day | 611 | 1.23 | 1.03, 1.47 | 0.020 | 1.22 | 1.02, 1.46 | 0.028 | 1.17 | 0.99, 1.41 | 0.086 |
| BMI= Body Mass Index, SSB= Sugar Sweetened Beverages, ASB= Artificially Sweetened Beverages  Logistic regression models. Outcome categories; BMI ≥30 (n=8358) vs. BMI<30 (n=18276)  Model 1: Adjusted for: age + sex  Model 2: model 1 + education + marital status  Model 3: Model 2 + smoking + alcohol consumption + physical activity + energy consumption + fruits and vegetables consumption + CVD or cancer in medical history + diabetes in medical history  p <0.05 was considered statistically significant | | | | | | | | | | | | |
